# Multi-donor multi-course faecal microbiota transplantation relieves the symptoms of chronic hemorrhagic radiation proctitis: A case report

**DOI:** 10.1097/MD.0000000000022298

**Published:** 2020-09-25

**Authors:** Ya-Mei Zheng, Xing-Xiang He, Harry Hua-Xiang Xia, Yu Yuan, Wen-Rui Xie, Jie-Yi Cai, Jia-Ting Xu, Li-Hao Wu

**Affiliations:** Department of Gastroenterology, The First Affiliated Hospital of Guangdong Pharmaceutical University, Guangzhou, Guangdong Province, China.

**Keywords:** chronic hemorrhagic radiation proctitis, faecal microbiota transplantation, intestinal bacteria

## Abstract

**Rationale:**

There are many treatments for chronic hemorrhagic radiation colorectal inflammation, but only a few treatments are supported by high-quality research evidence. Studies have shown that the occurrence and development of radiation proctitis are closely associated with the intestinal flora. Animal studies have indicated that faecal microbiota transplantation (FMT) can improve radiation enteropathy in a mouse model.

**Patient concerns:**

A 45-year-old female patient suffered from recurrent hematochezia and diarrhea for half a year after radiotherapy and underwent recurrent transfusion treatments. Colonoscopy showed obvious congestion of the sigmoid colon and rectal mucosa, a smooth surface, and bleeding that was easily induced by touch, which are consistent with radiation proctitis. The pathological findings revealed chronic mucosal inflammation. The magnetic resonance imaging examination of the pelvic cavity with a plain scan and enhancement showed changes after radiotherapy and chemotherapy, and no obvious tumor recurrence or metastasis was found. The laboratory examinations excluded pathogen infection.

**Diagnoses:**

Based on the history and examinations, the final diagnosis of this patient was chronic hemorrhagic radiation proctitis.

**Interventions:**

The patient was treated with a total of 4 individual courses of FMT.

**Outcomes:**

After the six-month follow-up, her hematochezia, abdominal pain and diarrhea were relieved. Furthermore, 16S rRNA sequencing of the feces showed that the intestinal bacterial composition of the patient obviously changed after FMT and became similar to that of the donors.

**Lessons:**

This case report shows that FMT can relieve the symptoms of hematochezia and diarrhea by changing the bacterial community structure in patients with chronic hemorrhagic radiation proctitis.

## 1 Introduction

The main clinical manifestations of chronic hemorrhagic radiation proctitis include hematochezia, diarrhea, tenesmus, rectal stricture, bowel fistula, and anal incontinence, and these symptoms usually occur several months after pelvic radiotherapy for pelvic cancer. The current treatments for chronic hemorrhagic radiation proctitis mainly include anti-inflammatory medications, antioxidants, probiotics, antibiotics, formalin, endoscopic therapy (endoscopic argon beam plasma coagulation), and surgery.^[1]^ These treatments have shown some efficacy, but few treatment methods are supported by high-quality research evidence.

In recent years, the relationships between intestinal bacteria and diseases have been increasingly revealed. Faecal microbiota transplantation (FMT) has shown good clinical efficacy in the treatment of various clinical diseases, such as refractory *Clostridium difficile* infection,^[2]^ irritable bowel syndrome,^[3]^ inflammatory bowel disease,^[4]^ and constipation.^[5]^ Animal studies have proven that FMT can improve radiation enteropathy in a mouse model by restoring the structural composition of the gut bacteria.^[6]^ In addition, a case report in which the donor was the patients son showed that radiation enteritis was alleviated by FMT.^[7]^ This paper reports a case of a chronic hemorrhagic radiation enteritis patient who was treated with multi-donor and multi-course FMT and achieved significant remission. The analysis of the gut microbiota of the patient and donors by 16S rRNA sequencing showed that compared to before the treatment, after the FMT treatment, the patients intestinal bacterial composition structure obviously changed and was similar to that of the donors.

## 2 Case presentation

### 2.1 Chief complaints and history of present illness

The patient was a 45-year-old female who was admitted to the Department of Gastroenterology on March 2, 2019 for “recurrent hematochezia for half a year after radiotherapy”. In 2017, due to cervical cancer, the patient underwent 28 radiotherapy treatments and 8 rounds of chemotherapy at a local hospital. During radiotherapy, diarrhea consisting of a black pasty stool occurred 7 to 8 times per day. After radiotherapy, the diarrhea resolved. Half a year ago (September 2018), the patient began to repeatedly experience mucus-containing bloody stool alternately with black pasty stool. The blood in the bloody stool was a bright red color accompanied by dark red clots, and mucus was present. The volume was approximately 30 to 50 ml occurring 5 to 6 times per day and approximately 2 to 3 days per week. The black pasty stool occurred 1 to 2 times per day with a volume of approximately 100 to 250 g each time and occurred approximately 2 to 3 days per week. The diarrhea was occasionally accompanied by abdominal pain and tenesmus, and there was no fever. Dizziness, fatigue, and polypnea gradually appeared after daily activities. The patient was admitted to a local hospital 3 times for severe anemia. The routine blood examination showed a minimum hemoglobin concentration of 30 g/L, and after transfusion, dizziness, polypnea, and fatigue improved. However, the above symptoms continued to repeatedly occur, and the patient sought further treatment at our hospital.

### 2.2 Physical examination upon admission

The patients temperature was 36.4°C, heart rate was 100 bpm, respiratory rate was 20 breaths per minute, blood pressure was 114/76 mm Hg and body mass index was 20.8 kg/m^2^. The patient presented an anemic face and a soft abdomen, and no mass was palpated. There was tenderness in the lower abdomen and no rebound pain. Bowel sounds occurred 5 times/minute.

### 2.3 Laboratory examination upon admission

A routine stool and faecal occult blood test showed a positive faecal occult blood test and a positive faecal fat test. The faecal pathogenic bacterial cultures showed fungal growth on March 2. However, there was no fungal growth on March 12, and we hypothesized that contamination was detected on March 2. The Epstein-Barr virus (EBV) antibody and DNA quantity, cytomegalovirus (CMV) antibody, *Clostridium. difficile* antigen, tuberculosis antibody, T cell spot test for tuberculosis infection, and coagulation function detection showed no abnormalities. The routine blood examinations showed a hemoglobin concentration of 53 g/L, indicating microcytic hypochromic anemia.

### 2.4 Imaging examinations

**Figure 1:**
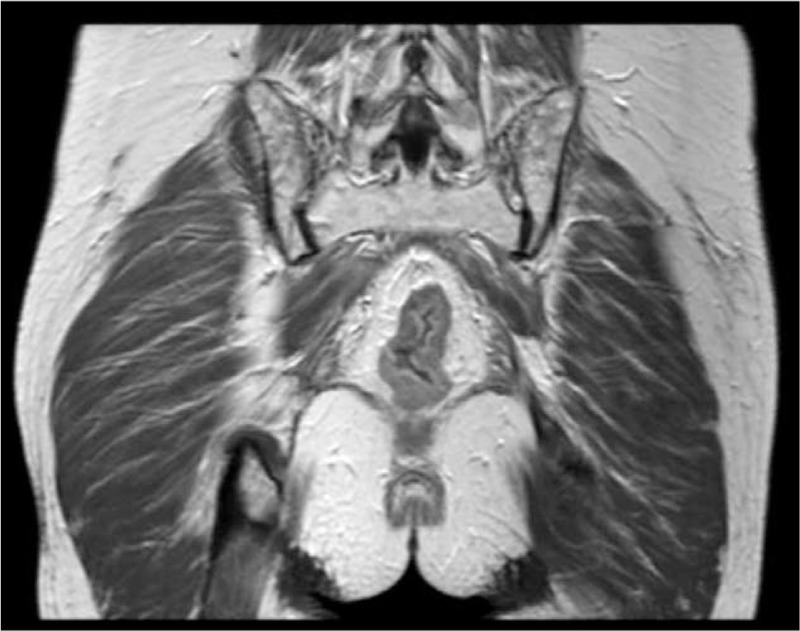
Magnetic resonance imaging examination of the patient before the treatment showed thickening and swelling of the colorectal wall.

**Figure 2:**
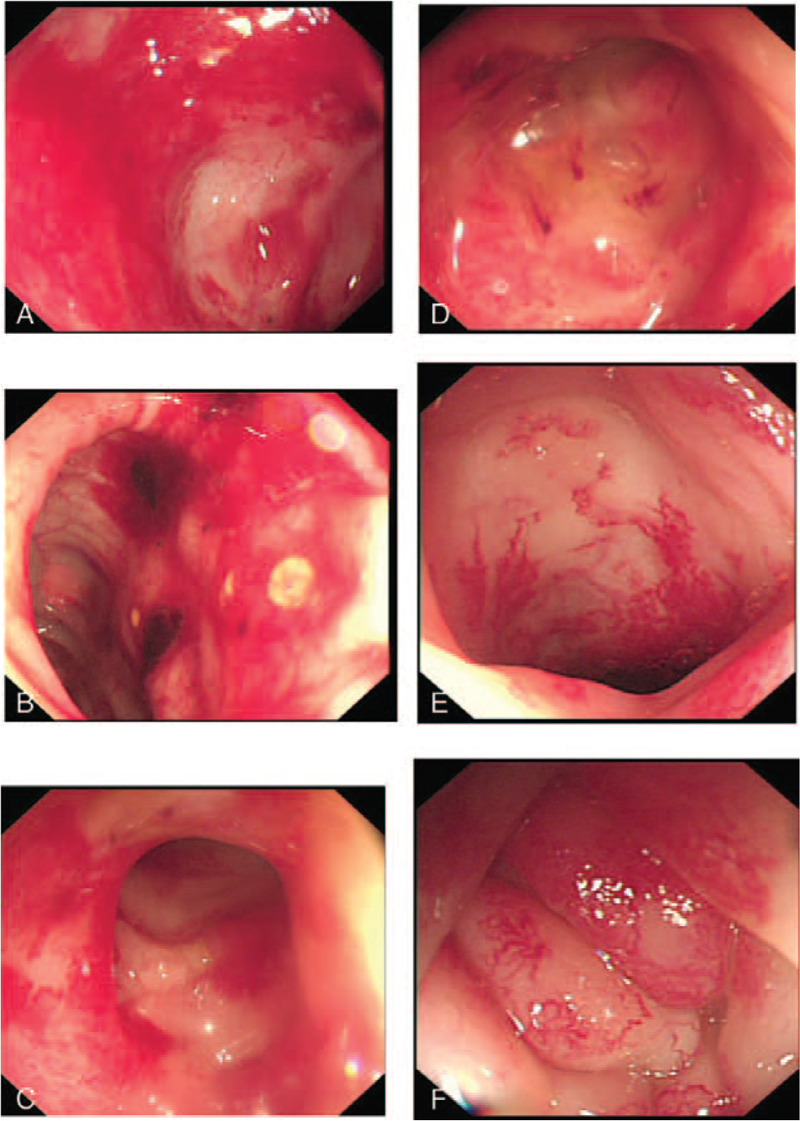
Colonoscopy showed mucosal inflammation of the sigmoid colon and rectum before the faecal microbiota transplantation and improvement in inflammation after the treatment with faecal microbiota transplantation (FMT). 2a-2b: colonoscopy before the treatment; 2c-2d: colonoscopy 4 weeks after the first course of FMT; 2e-2f: colonoscopy 4 weeks after the second course of FMT. These figures show that marked congestion and narrowing of the intestinal cavity at the sigmoid colon 20 cm from the anus occurred; thus, the passage of the colonoscope was difficult. Additionally, following the FMT treatment, the intestinal mucosal congestion was significantly improved.

**Figure 3:**
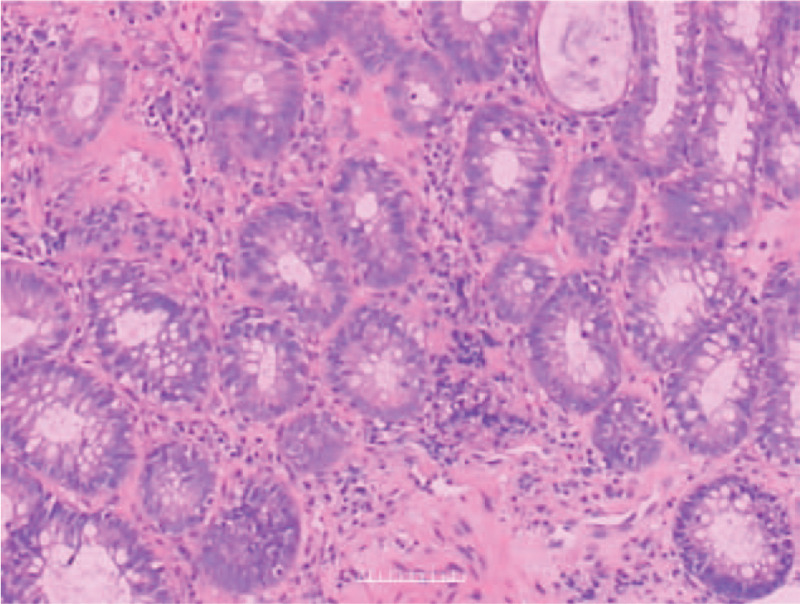
Rectal mucosal pathology of the patient before the treatment shows chronic mucosal inflammation with mild atypical hyperplasia of the glands.

The magnetic resonance imaging examination of the pelvic cavity with a plain scan and enhancement showed changes after radiotherapy and chemotherapy for cervical cancer, and no obvious tumor recurrence or metastasis was found in the pelvic cavity. The walls of the sigmoid colon and rectum were slightly thickened and swollen, which was considered to have occurred after radiotherapy (Fig. 1). Colonoscopy showed obvious congestion of the sigmoid colon and rectal mucosa and a smooth surface, and bleeding was easily induced by touch. The sigmoid colon was narrow, rendering the passage of the colonoscope difficult (Fig. 2a-2b). The colonoscopy diagnosis was radiation proctitis. The pathological findings revealed chronic mucosal inflammation with mild atypical hyperplasia of the glands (Fig. 3).

Based on the above examination results and after the exclusion of diseases, such as tumor recurrence, invasion of the rectum, fungal infection, *Clostridium difficile* infection, EBV or CMV infection, and ulcerative colitis, a diagnosis of “chronic hemorrhagic radiation proctitis” was rendered. Considering that many studies have reported that intestinal bacteria are involved in the incidence of radiation enteritis and that both animal model experiments and case reports have shown that FMT can improve the symptoms of radiation enteritis and mucosal inflammation,^[6–9]^ the patient and her family agreed to FMT treatment after being fully informed.

### 2.5 Faecal microbiota transplantation process

Faecal bacteria were obtained from 8 healthy people aged 21 to 24 years without digestive system diseases, tumors, infectious diseases, metabolic diseases, genetic diseases, or other related diseases after auxiliary examination. Additionally, the subjects did not take antibiotics, drugs affecting gastrointestinal dynamics or other drugs causing intestinal microecological disorders in the prior 3 months. Fresh faecal liquid was separated by an automatic purification system (GenFMTer; FMT Medical, Nanjing, China). Two FMT treatment approaches^[10]^ were utilized as follows: the lower-gut approach was adopted in the first course of treatment, and the mid-gut approach was utilized in the second to fourth courses of treatment because it was difficult to pass the endoscope through the narrow intestinal cavity. The faecal liquid was moved from the donor to the patient within 1 hour to keep it as fresh as possible to achieve maximum clinical efficacy. The regimen of 1 course of treatment was 200 ml/treatment/donor once a day for 3 days.

Treatment courses: The patient received a total of 4 individual courses of FMT, which were completed in March, April, May, and July.

This study was approved and monitored by the Ethics Committee of the First Affiliated Hospital of Guangdong Pharmaceutical University (Approval No. Yilun Shen [2019] No. 188).

### 2.6 Clinical efficacy and follow-up

At the six-month follow-up after all 4 courses of FMT were completed, hematochezia, abdominal pain and diarrhea were resolved, and the patient did not experience any side effects from the FMT treatment. Compared to her symptoms before the treatment, her stool frequency decreased from a maximum of 6 times to 2 times per day, and the mucus-containing bloody stool and pasty stool became soft yellow stool. Additionally, the European Organization for Research and Treatment of Cancer Therapy Oncology Group (RTOG/EORTC) score^[11]^ improved from level 2 to level 1. The patient was elated because she did not need a blood transfusion, proving that no blood was lost, and the hemoglobin concentration remained above 60 g/L (Table 1). The sigmoid colon and rectal mucosal inflammation showed improvement via colonoscopy (Fig. 2c-2f).

**Table 1:**
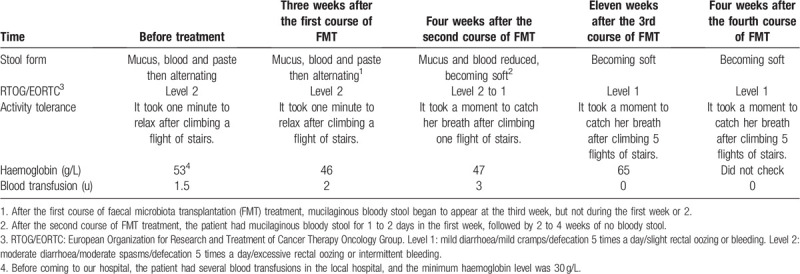
Patient disease changes over half a year.

**Figure 4:**
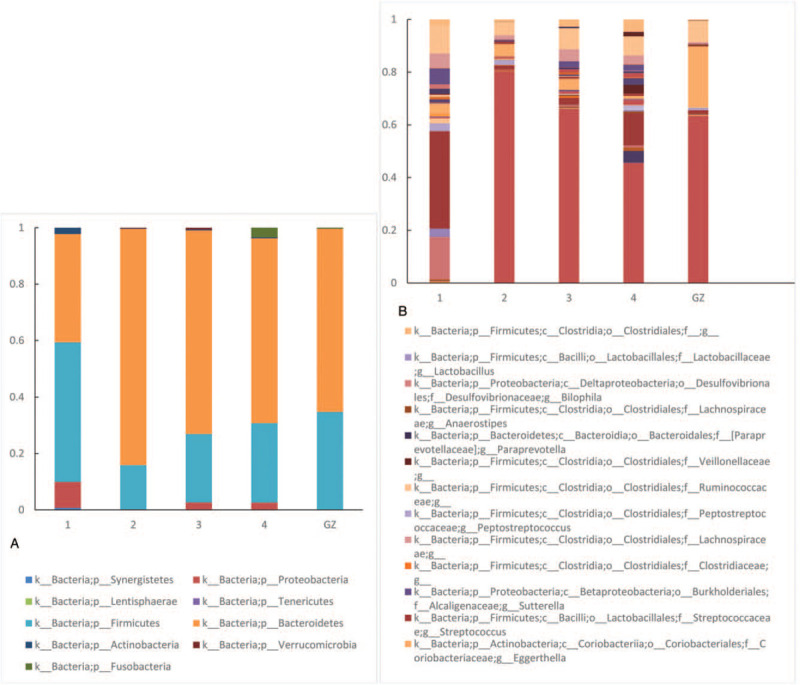
Distribution of the bacterial flora in the donor group and the patient before and after the faecal microbiota transplantation (FMT) treatment at the phylum and genus levels. 1, before the treatment; 2, 28 days after the first course of FMT; 3, 30 days after the second course of FMT; 4, 70 days after the third course of FMT; GZ, donor. 4a shows the changes in the bacterial flora in the donor group and the patient before and after the FMT treatment at the phylum level, whereas 4b shows the changes at the genus level.

The faecal samples collected from the patient before the treatment (1), 28 days after the first course of the FMT treatment (2), 30 days after the second course of the FMT treatment (3), and 70 days after the third course of treatment (4) and the donors (mix of the first course faecal liquid from 3 donors, GZ) were subjected to 16S rRNA sequencing. On average, 340.06 operational taxonomic units (OTUs) were identified with a minimum of 231 OTUs and a maximum of 500 OTUs. According to the analysis of the alpha diversity index, the species richness, evenness and inter-species differences significantly increased concurrently with the extension of time and increases in the transplantation time (Table 2). At the phylum level (Fig. 4a), the predominant bacteria were firmicutes before the transplantation (1) and bacteroidetes after the transplantation. However, at the genus level (Fig. 4b), the primary bacteria were bacteroidetes before the transplantation (1) and Prevotella after the transplantation. As the FMT course progressed, the composition of the microbiota of the patient tended to become similar to that of the donors.

**Table 2:**
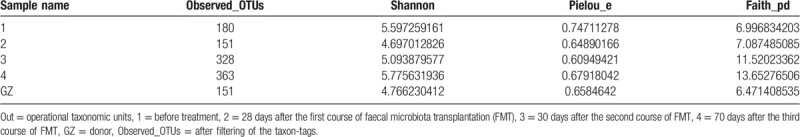
Statistical table of the alpha diversity index.

## 3 Discussion

In this case report, we presented a patient who developed chronic hemorrhagic radiation proctitis after chemoradiotherapy for cervical cancer and experienced relief of hematochezia after 4 courses of FMT treatment. The composition of the intestinal bacteria showed significant changes by 16S rRNA sequencing, indicating that FMT is effective for patients with chronic hemorrhagic proctitis.

Acute radiation proctitis occurs in more than 75% of patients who receive pelvic radiotherapy, and 5% to 20% of patients develop chronic radiation proctitis.^[1]^ The exposure of the intestinal tissue to radiation can induce apoptosis in epithelial cells, leading to mucosal inflammation, edema and ulceration, and subsequently to occlusive endarteritis, submucosal fibrosis and angiogenesis. Eventually, diarrhea, bleeding, pain and an impaired quality of life occur, representing the histopathological process of acute and chronic radiation proctitis.^[12]^ In this case, we show that colorectal inflammation improved after FMT. Thus, intestinal bacteria can improve proctitis.

Several studies have shown that the intestinal microbiota is associated with radiation-associated intestinal disease. One animal study showed that the survival rate of mice receiving a systemic lethal dose of radiation after antibiotic treatment was significantly higher than that of the control mice (after saline treatment), suggesting that the intestinal radiation sensitivity is related to the microbiota.^[6]^ Studies of radiation-induced proctitis in mice have shown that the composition of intestinal bacteria in the irradiated area changes, the diversity of firmicutes and bacteroidetes decreases, and the abundance of the proteus increases.^[8]^ The increased proteus abundance increases the expression of interleukin-1β and tumor necrosis factor-α in intestinal epithelial cells, suggesting that interleukin-1 is a major driver of tissue damage in radiation proctitis. In a study involving 9 gynecological tumor patients who received pelvic radiotherapy, the abundance of firmicutes and *Clostridium* was significantly reduced by 10% and 3%, respectively.^[9]^ Animal and human studies have shown that the diversity and composition of the intestinal flora significantly changes after radiotherapy. Intestinal microbiota may play a role by affecting and regulating oxidative stress, inflammatory processes, intestinal permeability, the composition of the mucous layer, epithelial repair, resistance to harmful stimuli, and the expression and release of immune molecules in the intestinal tract. Some studies used bacteria for the treatment of radiation proctitis. In 2014, a study showed that synbiotics could alleviate the symptoms of radiation proctitis and improve the quality of life of patients.^[13]^ The results showed that diarrhea decreased in patients treated with *Lactobacillus acidophilus* and *L. acidophilus* combined with *Bifidobacterium*. The Multinational Association of Supportive Care in Cancer and International Society of Oral Oncology guidelines support the use of probiotics to prevent diarrhea caused by radiotherapy.^[14]^ However, the results of a recent meta-analysis suggest that currently, evidence suggesting that probiotics are beneficial for preventing radiation-induced diarrhea is lacking.^[15]^ As symbiotic microbial flora, intestinal bacteria play an indispensable role in human health and diseases; thus, changing an individual bacterial species alone may not have an effect. FMT of the whole intestinal flora has been increasingly recognized as a treatment for diseases. Studies using mice have shown that FMT can reduce and protect against radiation-induced injury, improve the survival rate of irradiated mice, improve gastrointestinal functionality and epithelial integrity, and thus reduce radiation-induced gastrointestinal toxicity.

According to research, after radiotherapy, the microbiota diversity is decreased, with a high ratio of firmicutes to bacteroidetes increasing the likelihood of diarrhea.^[16]^ The symptoms of the patient were mainly characterized by diarrhea and blood, which obviously improved after the treatment with 4 courses of FMT. The endoscopic results showed that the colorectal mucous membrane inflammation was improved. The 16S rRNA sequencing showed an increase in the intestinal flora diversity and altered composition with a decreased abundance of firmicutes and an increased abundance of bacteroidetes, which is consistent with the results of the aforementioned research reports.^[16]^

In this case, a multi-donor, multi-course fresh faecal suspension for FMT was adopted. The purpose of multiple donors is to increase the diversity of the microbiota. The multi-course treatment is based on the reconstruction and consolidation of the intestinal bacterial balance in patients to maintain new intestinal bacterial homeostasis, consolidate the therapeutic effect, maintain clinical remission, and finally achieve a clinical cure. In this case, after 4 courses of FMT, the hematochezia and diarrhea disappeared, proving that FMT was effective in the treatment of chronic hemorrhagic radiation proctitis. However, we are uncertain which type of bacteria was responsible for the treatment effect. It is also possible that all bacteria worked together to produce the effect because symbiotic intestinal bacteria do not act alone. Additionally, studies investigating radiation-induced intestinal disease revealed that intestinal bacterial changes are different. Different studies of probiotic use for the treatment of radiation-induced intestinal disease have produced different or contrasting results. Therefore, we still conclude that the whole intestinal microbial flora plays a role in treatment. The increased microbial diversity may be the most obvious reason for the treatment efficacy.

## 4 Conclusion and prospects

This case report shows that FMT can relieve the symptoms of hematochezia and diarrhea in patients with chronic hemorrhagic radiation proctitis and that the bacterial community structure changes after treatment. FMT therapy may be a new and enhanced treatment for patients with radiation enteritis, which needs to be further confirmed by prospective and clinical trials involving large sample sizes.

## Author contributions

**Conceptualization:** Xing-xiang He, Jia-Ting Xu.

**Data curation:** Ya-Mei Zheng, Jie-Yi Cai.

**Formal analysis:** Yu Yuan, Wen-Rui Xie.

**Investigation:** Ya-Mei Zheng.

**Methodology:** Wen-Rui Xie.

**Validation:** Jia-Ting Xu.

**Visualization:** Harry Hua-Xiang Xia.

**Writing – original draft:** Ya-Mei Zheng.

**Writing – review & editing:** Harry Hua-Xiang Xia, Jia-Ting Xu.
